# Scalable Workflows and Reproducible Data Analysis for Genomics

**DOI:** 10.1007/978-1-4939-9074-0_24

**Published:** 2019-01-01

**Authors:** Francesco Strozzi, Roel Janssen, Ricardo Wurmus, Michael R. Crusoe, George Githinji, Paolo Di Tommaso, Dominique Belhachemi, Steffen Möller, Geert Smant, Joep de Ligt, Pjotr Prins

**Keywords:** Bioinformatics, Evolutionary biology, Big data, Parallelization, MPI, Cloud computing, Cluster computing, Virtual machine, MrBayes, Debian Linux, GNU Guix, Bioconda, CWL, Common Workflow Language, Guix Workflow Language, Snakemake, Nextflow

## Abstract

Biological, clinical, and pharmacological research now often involves analyses of genomes, transcriptomes, proteomes, and interactomes, within and between individuals and across species. Due to large volumes, the analysis and integration of data generated by such high-throughput technologies have become computationally intensive, and analysis can no longer happen on a typical desktop computer.

In this chapter we show how to describe and execute the same analysis using a number of workflow systems and how these follow different approaches to tackle execution and reproducibility issues. We show how any researcher can create a reusable and reproducible bioinformatics pipeline that can be deployed and run anywhere. We show how to create a scalable, reusable, and shareable workflow using four different workflow engines: the Common Workflow Language (CWL), Guix Workflow Language (GWL), Snakemake, and Nextflow. Each of which can be run in parallel.

We show how to bundle a number of tools used in evolutionary biology by using Debian, GNU Guix, and Bioconda software distributions, along with the use of container systems, such as Docker, GNU Guix, and Singularity. Together these distributions represent the overall majority of software packages relevant for biology, including PAML, Muscle, MAFFT, MrBayes, and BLAST. By bundling software in lightweight containers, they can be deployed on a desktop, in the cloud, and, increasingly, on compute clusters.

By bundling software through these public software distributions, and by creating reproducible and shareable pipelines using these workflow engines, not only do bioinformaticians have to spend less time reinventing the wheel but also do we get closer to the ideal of making science reproducible. The examples in this chapter allow a quick comparison of different solutions.

## Introduction

### 1.1 Overview

In this chapter, we show how to create a bioinformatics pipeline using four workflow systems: CWL, GWL, Snakemake, and Nextflow. We show how to put them together, so you can adapt it for your own purposes while discussing in the process the different approaches. All scripts and source code can be found on GitHub. The online material allows a direct comparison of how such workflows are assembled with their syntax.

Due to large volumes, the analysis and integration of data generated by high-throughput technologies have become computationally intensive, and analysis can no longer happen on a typical desktop computer. Researchers therefore are faced with the need to scale analyses efficiently by using high-performance compute clusters or cloud platforms. At the same time, they have to make sure that these analyses run in a reproducible manner. And in a clinical setting, time becomes an additional constraint, with motivation to generate actionable results within hours.

In the case of evolutionary genomics, lengthy computations are often multidimensional. Examples of such expensive calculations are Bayesian analyses, inference based on hidden Markov models, and maximum likelihood analysis, implemented, for example, by MrBayes [1], HMMER[2], and phylogenetic analysis by maximum likelihood (PAML) [3]. Genome-sized data, or Big Data [4, 5], such as produced by high-throughput sequencers, as well as growing sample size, such as from UK Biobank, the Million Veterans Program, and the other large genome-phenome projects, are exacerbating the computational challenges, e.g., [6].

In addition to being computationally expensive, many implementations of major algorithms and tools in bioinformatics do not scale well. One example of legacy software requiring lengthy computation is Ziheng Yang’s CodeML implementation of PAML [3]. PAML finds amino acid sites that show evidence of positive selection using *d*_N_/*d*_S_ ratios, i.e., the ratio of nonsynonymous and synonymous substitution rate. For further discussion see also Chapter. 12. Executing PAML over an alignment of 100 sequences may take hours, sometimes days, even on a fast computer. PAML (version 4.x) is designed as a single-threaded process and can only exploit one central processing unit (CPU) to complete a calculation. To test hundreds of alignments, e.g., different gene families, PAML is invoked hundreds of times in a serial fashion, possibly taking days on a single computer. Here, we use PAML as an example, but the idea holds for any software program that is CPU bound, i.e., the CPU speed determines program execution time. A CPU bound program will be at (close to) 100% CPU usage. Many legacy programs are CPU bound and do not scale by themselves.

Most bioinformatics (legacy) programs today do not make effective use of multi-core computers

The reason most bioinformatics software today does not make full use of multicore computers or GPUs is because writing such software is difficult. (See also the text box below for a further treatment of this topic; *see* Box 1.)

A common parallelization strategy in bioinformatics is to start with an existing nonparallel application and run it by dividing data into independent units of work or jobs which run in parallel and do not communicate with each other. This is also known as an “embar rassingly parallel“ solution, and we will pursue this below.

### 1.2 Parallelization in the Cloud

Cloud computing allows the use of “on-demand” CPUs accessible via the Internet and is playing an increasingly important role in bioinformatics. Bioinformaticians and system administrators previously had to physically install and maintain large compute clusters to scale up computations, but now cloud computing makes it possible to rent and access CPUs, GPUs, and storage, thereby enabling a more flexible concept of on-demand computing [7]. The cloud scales and commoditizes cluster infrastructure and management and, in addition, allows users to run their own operating system, usually not true with existing cluster and GRID infrastructure (a GRID is a heterogeneous network of computers that act together). A so-called hypervisor sits between the host operating system and the guest operating system, and it makes sure they are clearly separated while virtualizing host hardware. This means many guests can share the same machine that appears to the users as a single machine on the network. This allows providers to efficiently allocate resources. Containers are another form of light virtualization that is now supported by all the main cloud providers, such as Google, Microsoft, Rackspace OpenStack, and Amazon (AWS). Note that only OpenStack is available as free and open-source software.

An interesting development is that of portable batch systems (PBS) in the cloud. PBS-like systems are ubiquitous in high-performance computing (HPC). Both Amazon EC2 and Microsoft Cloud offer batch computing services with powerful configuration options to run thousands of jobs in the cloud while transparently automating the creation and management of virtual machines and containers for the user. As an alternative, Arvados is an open-source product specifically aimed at bioinformatics applications that makes the cloud behave as if it is a local cluster of computers, e.g., [8].

At an even higher level, MapReduce is a framework for distributed processing of huge datasets, and it is well suited for problems using large number of computers [9]. The map step takes a dataset and splits it into parts and distributes them to worker nodes. Worker nodes can further split and distribute data. At the reduce step, data is combined into a result, i.e., it is an evolved scatter and gather approach. An API is provided that allows programmers to access functionality. The Apache Hadoop project includes a MapReduce implementation and a distributed file system [10] that can be used with multiple cloud providers and also on private computer clusters. Another similar example is the Apache Spark project based on resilient distributed datasets (RDD)—a fault-tolerant collection of elements that can be accessed and operated on in parallel.

The advantage of such higher-level systems is that they go well beyond hardware virtualization: not only the hardware infrastructure but also the operating system, the job scheduler, and resource orchestration are abstracted away. This simplifies data processing, parallelization, and the deployment of virtual servers and/or containers. The downside is that users have less control over the full software stack and often needs to program and interact with an application programmers interface (API).

Overall, in the last decade, both commercial and noncommercial software providers have made cloud computing possible. Bioinformaticians can exploit these services.

### 1.3 A Pipeline for the Cloud

To create a bioinformatics pipeline, it is possible to combine remote cloud instances with a local setup. Prepare virtual machines or containers using similar technologies on a local network, such as a few office computers or servers, and then use these for calculations in the cloud when an analysis takes too long. The cloud computing resources may, for instance, support a service at peak usage, while regular loads are met with local infrastructure (i.e., burst compute). New ideas can be developed and pre-evaluated using modest in-house setups and then scaled to match the most demanding work.

Cloud services can be used for burst computing – enabling local clusters to be much smaller – as small as a single computer

In the following sections, we will provide instructions to deploy applications, and we will show how the use of workflow systems and reproducible environments can greatly simplify running scalable workflows on different environments, including the cloud.

### 1.4 Parallelization of Applications Using a Workflow

In case of embarrassingly parallel applications, programs are run independently as separate processes which do not communicate with each other. This is also a scatter and gather approach, i.e., inputs split into several jobs are fed into each process by the user. Job outputs are collected and collated. In bioinformatics, such tasks are often combined into computational pipelines. With the PAML example, each single job can be based on one alignment, potentially giving linear speed improvements by distributing jobs across multiple CPUs and computers. In other words, the PAML software, by itself, does not allow calculations in parallel, but it is possible to parallelize multiple runs of PAML by splitting the dataset. The downside of this approach is the deployment and configuration of pipeline software, as well as the management and complexity of splitting inputs and the collecting and collating of outputs. Also, pipelines are potentially fragile, because there is no real interprocess communication. For example, it is hard to predict the consequences of a storage or network error in the middle of a week- or month-long calculation.

Even for multithreaded applications that make use of multiple CPUs, such as BLAST and MrBayes, it is possible to scale up calculations by using a workflow. For example, MrBayes-MPI version 3.1.2 does not provide between-machine parallelization and is therefore machine bound, i.e., the machine’s performance determines the total run time. Still, if one needs to calculate thousands of phylogenetic trees, discrete jobs can be distributed across multiple machines. A similar approach is often used for large-scale BLAST analyses over hundreds of thousands of sequences.

A pipeline typically consists of linear components, where one software tool feeds into another, combined with a scattering of jobs across nodes and a gathering and collation of results.

In existing compute clusters, to distribute work across nodes, portable batch system (PBS) schedulers are used, such as Slurm [11]. Many pipelines in bioinformatics are created in the form of Bash, Perl, or Python scripts that submit jobs to these schedulers. Such scripted pipelines have the advantage that they are easy to write and adaptable to different needs. The downside is that they are hard to maintain and not very portable, since the description of the environment and the software packages are not part of these scripts, reducing or completing preventing the reproducibility of a certain analysis in a different context. This has led to the current state of affairs in bioinformatics that it is surprisingly hard to share pipelines and workflows. As a result much effort is spent reinventing the wheel.

Most existing bioinformatics pipelines cannot easily be shared and reproduced

In recent years, a number of efforts have started to address the problem of sharing workflows and making analyses reproducible. One example is the Common Workflow Language (CWL), a specification for describing analysis workflows and tools in a way that makes them portable and scalable across a variety of environments—from workstations to cluster, cloud, and HPC environments. CWL is a large bioinformatics community effort. Different platforms support CWL, including Arvados, Galaxy, and Seven Bridges [8].

A second workflow language is the Guix Workflow Language (GWL) built on top of the GNU Guix software deployment system. GWL aims to provide a deterministic and bit-reproducible analysis environment.

A third workflow language and orchestrator, Nextflow, allows scalable and reproducible scientific workflows to run seamlessly across multiple platforms from local computers to HPC clusters and the cloud, offering a concise and expressive DSL to describe complex workflows. Nextflow is routinely used in organizations and institutes, such as the Roche Sequencing, the Wellcome Trust Sanger Institute, and the Center for Genomic Regulation (CRG) Nextflow workshop.

Forth there is Snakemake, another widely used workflow manager system, written in Python and inspired by GNU Make. It allows for the composition of workflows based on a graph of rules whose execution is triggered by the presence, absence, or modification of expected files and directories.

It is interesting to note that all these workflow languages and systems originated in bioinformatics. It suggests that in this rapidly growing field, the increasing computational needs and moreover the diverse demands made more formal solutions a necessity. It also suggests that existing workflow engines used in astronomy and physics, for example, have different requirements.

Writing programs that fully utilize multi-core architectures is hard

Not only is parallel programming intrinsically complicated; programmers also have to deal with communication overheads between parallel threads. MrBayes, for example, a program for calculating phylogenetic trees based on Bayesian analysis, comes with MPI support. MPI is a message-based abstraction of parallelization, in the form of a binary communication protocol implemented in a C programming library [12]. In some cases the parallelized version is slower than the single CPU version. For example, the MPI version calculates each Markov chain in parallel, and the chains need to be synchronized with each other, in a “scatter and gather” pattern. The chains spend time waiting for each other in addition to the communication overheads introduced by MPI itself. Later MrBayes adopted a hybrid use of coarsegrained OpenMPI and fine-grained use of pthreads or OpenMP leading to improved scalability, e.g., [13].

Another example of communication overhead is with the statistical programming language R [14], which does not have native threading support built into the language. One possible option is to use an MPI-based library which only allows coarse-grained parallelization from R, as each parallelized R thread starts up an R instance, potentially introducing large overheads, both in communication time and memory footprint. For a parallelized program to be faster than its single-threaded counterpart, these communication overheads have to be dealt with.

Parallelization in R is coarse-grained with large overhead

The need for scaling up calculations on multi-CPU computers has increased the interest in a number of functional programming languages, such as Erlang [15], Haskell [16], Scala [17], and Julia [18]. These languages promise to ease writing parallel software by introducing a higher level of abstraction of parallelization, combined with immutable data, automatic garbage collection, and good debugging support [5, 19]. For example, Erlang and Scala rely on Actors as an abstraction of parallelization and make reasoning about fine-grained parallelization easier and therefore less error prone.

Actors were introduced and explored by Erlang, a computer language originally designed for highly parallelized telecommunications computing. To the human programmer, each Actor appears as a linear piece of programming and is parallelized without the complexity of locks, mutexes, and semaphores. Actors allow for parallelization in a manageable way, where lightweight threads are guaranteed to be independent and each has a message queue, similar to MPI. Actors, however, are much faster, more intuitive, and, therefore, probably, safer than MPI. Immutable data, when used on a single multi-CPU computer, allows fast passing of data by reference between Actors. When a computer language supports the concept of immutability, it guarantees data is not changed between parallel threads, again making programming less error prone and easier to structure. Actors with support for immutable data are implemented as an integral part of the programming language in Erlang, Haskell, Scala, Elixir, and D [20].

Another abstraction of parallelized programming is the introduction of goroutines, part of the Go programming language [21]. Where MPI and Actors are related to a concept of message passing and mail boxes, goroutines are more closely related to Unix named pipes. Goroutines also aim to make reasoning about parallelization easier, by providing a pipe where data goes in and results come out, and this processing happens concurrently without use of mutexes, making it easier to reason about linear code. Goroutines are related to communicating sequential processes (CSP), the original paper by Tony Hoare in 1978 [22]. Meanwhile, recent practical implementations are driven by the ubiquity of cheap multicore computers and the need for scaling up. A Java implementation of CSP exists, named JCSP [23], and a Scala alternative named CSO [24]. Go made goroutines intuitive and a central part of the strongly typed compiled language.

Erlang, Elixir, Haskell, Scala, Julia, Go and D are languages offering useful abstractions and tools for multi-core programming

It is important to note that the problems, ideas, and concepts of parallel programming are not recent. They have been an important part of computer science theory for decennia. We invite the reader interested in parallel programming to read up on the languages that have solid built-in support high-level parallelization abstractions, in particular, Scala [17], Go [21], and D [20].

#### 1.4.1 GPU Programming

Another recent development is the introduction of GPU computing or “heterogeneous computing” for offloading computations. Most GPUs consist of an array of thousands of cores that can execute similar instructions at the same time. Having a few thousand GPU cores can speed up processing significantly. Programming GPUs, however, is a speciality requiring specialized compilers and communication protocols, and there are many considerations, not least the I/O bottleneck between the main memory and the GPU’s dedicated RAM [5]. Even so, it is interesting to explore the use of GPUs in bioinformatics since they come with almost every computer today and clusters of GPU can increasingly be found in HPC infrastructure and in the cloud, alike. With the advent of “deep neural networks” and the general adoption of machine learning techniques for Big Data, GPUs have become a mainstream technology in data mining.

## 2 Package Software in a Container

Container technologies, such as Docker and Singularity, have gained popularity because they have less overhead than full virtual machines (VMs) and are smaller in size [24]. Containers are fully supported by the major cloud computing providers and play an important role for portability across different platforms.

Adoption of container solutions on HPC has been problematic, mostly because of security concerns. Singularity [26] offers a decentralized environment encapsulation that works in user space and that can be deployed in a simpler way since no root privileges are required to execute tools provided with Singularity. That is, Singularity containers can be created on a system with root privileges but run on a system without root privileges—though it requires some special kernel support. Docker containers can be imported directly in Singularity, so when we present how to build Docker container images in the following sections, the reader should be aware that the same images can also be used with Singularity. Singularity is slowly being introduced in HPC setups [27].

GNU Guix also has support for creating and running Linux containers. One interesting benefit is that, because the software packaging system is read-only and provides perfect isolation, containers automatically can share specific software running on the underlying system, making running containers even lighter and extremely fast.

In this section we discuss three popular software distribution systems for Linux: Debian GNU/Linux (Debian), GNU Guix, and Conda can be used together on a single system allowing access to most bioinformatics software packages in use today. In this section we bundle tools that can be deployed in a Docker image, which can run on a single multicore desktop computer and a compute cluster and in the cloud.

### 2.1 Debian Med

Debian (http://www.debian.org) is the oldest software distribution (started 1993) mentioned here with the largest body of software packages. Debian targets a wide range of architectures and includes a kernel plus a large body of other user software including graphical desktop environments, server software, and specialist software for scientific data processing. Overall Debian represents millions of users and targets most platforms in use today, even though it is not the only packaging system around (RPM being a notable alternative, for RedHat, Fedora, OpenSuSE, and CentOS).

Debian Med is a project within Debian that packages software for medical practice and biomedical research. The goal of Debian Med is a complete open system for all tasks in medical care and research [28]. With Debian Med over 400 precompiled bioinformatics software programs are available for Linux, as well as some 400 R packages. Proper free and open-source software (FOSS) can easily be packaged and distributed through Debian. Debian and its derivatives, such as Ubuntu and Mint, share the deb package format and have a long history of community support for bioinformatics packages [28, 29].

#### 2.1.1 Create a Docker Image with Debian

Using the bio packages already present in Debian, it is straightforward to build a Docker container that includes all the necessary software to run the example workflows. Here is the code for creating the Docker image ***(see*** also [30]). We created a pre-built Docker image which is available on Docker Hub [31].

Essentially, write a Docker script:

FROM debian:buster

RUN apt-get update && apt-get -y install perl clustalo paml

ADD pal2nal.pl /usr/local/bin/pal2nal.pl

RUN chmod +x /usr/local/bin/pal2nal.pl

And build and run the container:

docker build -t scalability_debian -f Dockerfile.debian

### 2.2 GNU Guix

GNU Guix (https://www.gnu.org/software/guix/) is a package manager of the GNU project that can be installed on top of other Linux distributions and represents a rigorous approach toward dependency management [32]. GNU Guix software packages are uniquely isolated by a hash value computed over all inputs, including the source package, the configuration, and all dependencies. This means that it is possible to have multiple versions of the same software and even different variants or combinations of software, e.g., Apache web server with SSL and without SSL compiled on a single system.

As of November 2017, GNU Guix provides over 6500 software packages, including a wide range of dedicated scientific software for bioinformatics, statistics, and machine learning

#### 2.2.1 Create a Docker Image with GNU Guix

GNU Guix has native support for creating Docker images. Creating a Docker image with GNU Guix is a one liner:

guix pack -f docker -S /bin=bin paml clustal-omega

which creates a reproducible Docker image containing PAML and Clustal Omega [33], including all of their runtime dependencies. Guix makes it very easy to write new package definitions using the Guile language (a LISP). If you want to include the definition of your own packages (that are not in Guix main line), you can include them dynamically. This is how we add pal2nal [34] in below GWL workflow example (see Subheading 3.3 below).

### 2.3 Conda

Conda (https://conda.io/docs/) is a cross-platform package manager written in Python that can be used to install software written in any language. Conda allows the creation of separate environments to deploy multiple or conflicting packages versions, offering a means of isolation. Note that this isolation is not as rigorous as that provided by GNU Guix or containers. The Bioconda [35] (https://bioconda.github.io/) project provides immediate access to over 2900 software packages for bioinformatics, and it is maintained by an active community of more than 200 contributors.

#### 2.3.1 Create a Docker Image with Bioconda

A Docker container can be created starting from the “Miniconda” image template, which is based on Debian. The Docker instructions are comparable to those of Debian above:

FROM conda/miniconda3

RUN conda config --add channels conda-forge

RUN conda install -y perl=5.22.0

RUN conda install -y -c bioconda paml=4.9 clustalo=1.2.4

wget=1.19.1

ADD pal2nal.pl /usr/local/bin/pal2nal.pl

RUN chmod +x /usr/local/bin/pal2nal.pl

Note that we provide the version numbering of the packages. If you want to build this container, you can use the Dockerfile provided in the GitHub repository [30] and then run:

docker build -t scalability.

We also added a pre-built container image on Docker Hub [31].

Conda can also be used outside any container system to install the software directly on a local computer or cluster. To do that first install the Miniconda package https://conda.io/miniconda.html, and then you can create a separate environment with the necessary software to run the workflows. Following is an example to set up a working environment:

conda create -n scalability

source activate scalability

conda config --add channels conda-forge

conda install -y perl=5.22.0

conda install -y -c bioconda paml=4.9 clustalo=1.2.4

wget=1.19.1

wget http://www.bork.embl.de/pal2nal/distribution/pal2nal.v14.tar.gz

tar xzvf pal2nal.v14.tar.gz

sudo cp pal2nal.v14/pal2nal.pl /usr/local/bin

sudo chmod +x /usr/local/bin/pal2nal.pl

Note that we use Miniconda here to bootstrap Bioconda. Bioconda can be bootstrapped in other ways. One of them is GNU Guix which contains a Conda package.

### 2.4 A Note on Software Licenses

All above packaging systems use free and open-source software (FOSS) released under a permissible license, i.e., a license permitting the use, modification, and distribution of the source code for any purpose. This is important because it allows software distributions to distribute all included software freely. Software that is made available under more restrictive licenses, such as for “academic nonprofit use only,” cannot be distributed in this way. An example is PAML that used to have such a license. Only when it was changed PAML got included into Debian, etc. Also, for this book chapter, we asked the author of pal2nal to add a proper license. After adding the GPLv2, it became part of the Debian distribution; see also https://tracker.debian.org/pkg/pal2nal. This means that above Docker scripts can be updated to install the pal2nal Debian package.

When you use scientific software, always check the type of license under which it is provided, to understand what you can or cannot do with it. When you publish software, add a license along with your code, so others can use it and distribute it.

Typical licenses used in bioinformatics are MIT (Expat) and BSD, which are considered very permissive, and also GPL and the Apache License, which are designed to grant additional protections with regard to derivative works and patentability. Whenever possible, free software licenses such as mentioned above are encouraged for scientific software. Check the guidelines of your employer and funding agencies.

## 3 Create a Scalable and Reusable Workflow

### 3.1 Example Workflow

We have created a number of examples to test a scalable and reproducible workflow, the full code, and examples that are available on GitHub [30]. In this case putative gene families of the oomycete *Phytophthora infestans* are tested for evidence of positive selection. *P. infestans* is a single-cell pathogen, which causes late blight of potato and tomato. Gene families under positive selection pressure may be involved in protein-protein interactions and are potentially of interest for fighting late blight disease.

As an example the P. *infestans* genome data [36] was fetched from http://www.broadinstitute.org/annotation/genome/phytophthora_infestans/MultiDownloads.html, and predicted genes were grouped by \name{blastclust} using 70% identity (see also Chapter. 21). This resulted in 72 putative gene families listed on the online repository on GitHub [30].

The example workflow aligns amino acid sequences using Clustal Omega, creates a neighbor joining tree, and runs CodeML from the PAML suite. The following is one example to look for evidence of positive selection in a specific group of alignments:

clustalo -i data/clusterXXXXX/aa.fa --guidetree-out=data/ clusterXXXXXX/aa.ph > data/clusterXXXXXX/aa.aln

pal2nal.pl -output paml data/clusterXXXXX/aa.aln data/clusterXXXXX/nt.fa > data/clusterXXXXX/alignment.phy

cd data/clusterXXXXX

Codeml ../paml0-3.ctl

First we align amino acid with Clustal Omega, followed by translation to a nucleotide alignment with pal2nal. Next we test for evidence of positive selection using PAML’s \name{Codeml} with models M0-M3. Note that the tools and settings used here are merely chosen for educational purposes. The approach itself here may result in false positives, as explained by Schneider et al. [37]. Also, PAML is not the only software that can test for evidence of positive selection, for example, the HyPhy molecular evolution and statistical sequence analysis software package contains similar functionality and uses MPI to parallelize calculations [38]. PAML is used here because it is a reference implementation and is suitable as an example how a legacy single-threaded bioinformatics application can be parallelized in a workflow.

In the next section, different workflow systems are presented that can be used to run the described analysis: in a scalable and reproducible manner, locally on a desktop, on a computer cluster, or in the cloud. All the code and data to run these examples is available on GitHub [30]. To load the code on your desktop, clone the git repository locally. The examples can be executed from the repository tree:

git clone https://github.com/EvolutionaryGenomics/scalabil-ity-reproducibility-chapter.git

### 3.2 Common Workflow Language

Common workflow language (CWL, http://www.commonwl.org/) is a standard for describing workflows that are portable across a variety of computing platforms [39]. CWL is a specification and not a software in itself though it comes with a reference implementation which can be run with Docker containers. CWL promotes an ecosystem of implementations and supporting systems to execute the workflows across multiple platforms. The promise is that when you write a workflow for, e.g., Arvados, it should also run on another implementation, e.g., Galaxy.

Given that CWL takes inspiration from previously developed tools and GNU Make in particular [40], the order of execution in a CWL workflow is based on dependencies between the required tasks. However unlike GNU Make, CWL tasks are defined to be isolated, and you must be explicit about inputs and outputs. The benefits of explicitness and isolation are flexibility, portability, and scalability: tools and workflows described with CWL can transparently leverage software deployment technologies, such as Docker, and can be used with CWL implementations from different vendors, and the language itself can be applied to describe large-scale workflows that run in HPC clusters, or the cloud, where tasks are scheduled in parallel across many nodes.

CWL workflows are written in JSON or YAML formats. A workflow consists of blocks of steps, where each step in turn is made up of a task description that includes the inputs and outputs of the task itself. The order of execution of the tasks is determined automatically by the implementation engine. In the GitHub repository, we show an example of a CWL workflow to describe the analysis over the protein alignments. To test the workflow, you will need the CWL reference runner implementation:

pip install cwlref-runner

and then to run the example from the repository tree:

CWL/workflow.cwl --clusters data

To run the CWL workflow on a grid or cloud multi-node system, we can install another CWL implementation, this one built upon the toil platform [41]:

pip install toil[cwl]

toil-cwl-runner CWL/workflow.cwl --clusters data

**Fig. 1:**
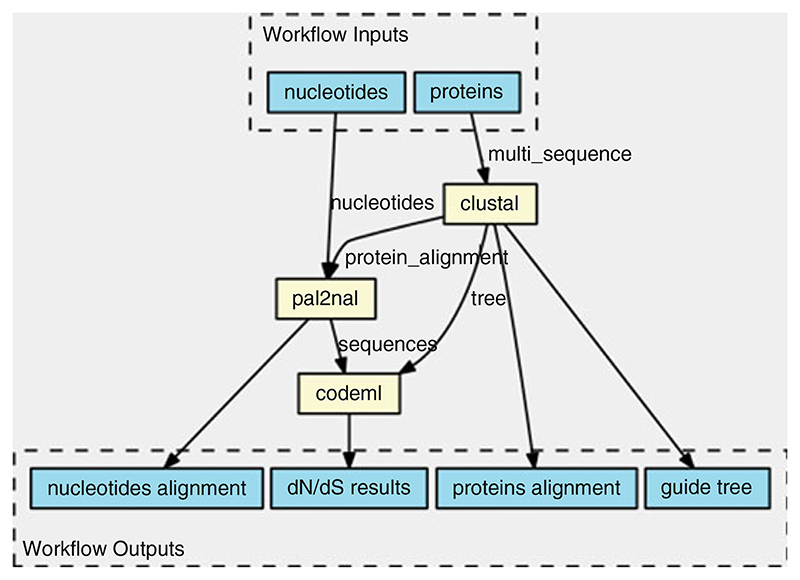
Workflow automatically generated from the CWL schema displays how PAML’s Codeml receives inputs from two sources and outputs the *d*_N_/*d*_S_ information. A workflow engine figures out that it has to run clustal first, followed by pal2nal and Codeml as a linear sequence. For each input, the job can be executed in parallel

CWL comes with extra tooling, such as visualization of CWL workflows (Fig. 1).*See* view.commonwl.org for more examples.

### 3.3 Guix Workflow Language

The Guix Workflow Language (GWL) extends the functional package manager GNU Guix [32] with workflow management capabilities. GNU Guix provides an embedded domain-specific language (EDSL) for packages and package composition. GWL extends this EDSL with processes and process composition.

In GWL, a process describes the computation, for example, running the clustalo program. A workflow in the GWL describes how processes relate to each other. For example, the Codeml program can only run after both clustalo and pal2nal finished successfully.

The tight coupling of GWL and GNU Guix ascertains that not only the workflow is described rigorously but also the deployment of the programs on which the workflow depends.

To run the GWL example, you need to install GNU Guix (https://www.gnu.org/software/guix/manual/html_node/Binary-Installation.html) and the GWL installed on your computer. Once GNU Guix is available, installing GWL can be done using:

guix package -i gwl

The example can be run using:

cd scalability-reproducibility-chapter/GWL

guix workflow -r example-workflow

GWL also implements execution engines to offload computation on compute clusters, allowing it to scale. The process engines can use the package composition capabilities of GNU Guix to create the desirable form of software deployment—be it installing programs on the local computer or creating an application bundle, a Docker image, or a virtual machine image.

Running our example on a cluster that has Grid Engine:

guix workflow -r example-workflow -e grid-engine

GNU Guix + GWL can ensure full reproducibility of an analysis, including all software dependencies—all the way down to glibc. GNU Guix computes a unique string, a hash, on the complete set of inputs and the build procedure of a package. It can guarantee that a package is built with the same source code, dependency graph, and the same build procedure, and produces identical output. In GWL for each process and workflow, a hash is computed of the packages, the procedure, and the execution engine. By comparing hashes it is not only possible to compare whether the workflow is running using the exact same underlying software packages, and using the same procedures, but also the full graph of dependencies can be visualized. To obtain such an execution plot:

guix package -i graphviz

guix workflow -g example-workflow | dot -Tpdf > example- workflow.pdf

Note that, unlike the other workflow solutions discussed here, GWL does not use the time stamps of output files. The full dependency graph is set before running the tools, and it only needs to check whether a process returns an error state. This means that there are no issues around time stamps and output files do not have to be visible to the GWL engine.

### 3.4 Snakemake

Snakemake [42] is a workflow management system that takes inspiration from GNU Make [40], a tool to coordinate the compilation of large programs consisting of interdependent source files (https://snakemake.readthedocs.io/en/stable/).

Snakemake provides a DSL that allows the user to specify generator rules. A rule describes the steps that need to be performed to produce one or more output files, such as running a shell script. These output files may be used as inputs to other rules. The workflow is described as a graph in which the nodes are files (provided input files, generated intermediate files, or the desired output files) and the edges are inferred from the input/output interdependencies of connected rules.

When a user requests a certain file to be generated, Snakemake matches the file name against concrete or wildcard rules, traverses the graph from the target file upward, and begins processing the steps for every rule for which no new output file is available. Whether or not an output file is considered new depends on its time stamp relative to the time stamp of prerequisite input files. In doing so, Snakemake only performs work that has not yet been done or for which the results are out of date, just like GNU Make. Snakemake can be configured to distribute jobs to batch systems or to run jobs on the local system in parallel. The degree of parallelization depends on the dependencies between rules.

Snakemake is written in Python and allows users to import Python modules and use them in the definition of rules, for example. It also has special support for executing R scripts in rules, by exposing rule parameters (such as inputs, outputs, concrete values for wildcards, etc.) as an S4 object that can be referenced in the R script.

Snakemake provides native support for the Conda package manager. A rule may specify a Conda [35] environment file describing a software environment that should be active when the rule is executed. Snakemake will then invoke Conda to download the required packages as specified in the environment file. Alternatively, Snakemake can interface with an installation of the Singularity container system [26] and execute a rule within the context of a named application bundle, such as a Docker image.

To run the Snakemake workflow, you need to install Snakemake (example showed with Conda):

conda install -y -c bioconda snakemake=4.2.0

And then to run the example from the repository tree:

cd Snakemake

snakemake

### 3.5 Nextflow

Nextflow [43] is a framework and an orchestration tool that enables scalable and reproducible scientific workflows using software containers (https://www.nextflow.io/). It is written in the Groovy JVM programming language [44] and provides a domain-specific language (DSL) that simplifies writing and deploying complex workflows across different execution platforms in a portable manner.

A Nextflow pipeline is described as a series of processes, where each process can be written in any language that can be executed or interpreted on Unix-like operating systems (e.g., Bash, Perl, Ruby, Python, etc.). A key component of Nextflow is the dataflow programming model, which is a message-based abstraction for parallel programming similar to the CSP paradigm (*see* [23]). The main difference between CSP and dataflow is that in the former, processes communicate via synchronous messages, while in the latter, the messages are sent in an asynchronous manner. This approach is useful when deploying large distributed workloads because it has latency tolerance and error resilience. In practical term the dataflow paradigm uses a push model in which a process in the workflow sends its outputs over to the downstream processes that waits for the data to arrive before starting their computation. The communication between processes is performed through channels, which define inputs and outputs for each process. Branches in the workflow are also entirely possible and can be defined using conditions that specify if a certain process must be executed or not depending on the input data or on user defined parameters.

The dataflow paradigm is the closest representation of a pipeline idea where, after having opened the valve at the beginning, the flow progresses through the pipes. But Nextflow can handle this data flow in a parallel and asynchronous manner, so a process can operate on multiple inputs and emit multiple outputs at the same time. In a simple workflow where, for instance, there are 100 nucleotide sequences to be aligned with the NCBI NT database using BLAST, a first process can compute the alignment of the 100 sequences independently and in parallel, while a second process will wait to receive and collect each of the outputs from the 100 alignments to create a final results file. To allow workflow portability, Nextflow supports multiple container technologies such as Docker and Singularity and integrates natively with Git and popular code sharing platforms, such as GitHub. This makes it possible to precisely prototype self-contained computational workflows, tracking also all the modifications over time and ensuring the reproducibility of any former configuration. Nextflow allows executing workflows across different computing platforms by supporting several cluster schedulers (e.g., SLURM, PBS, LSF and SGE) and allowing direct execution on the Amazon cloud (AWS), using services, such as AWS Batch or automating the creation of a compute cluster in the cloud for the user.

To run the Nextflow example, you need to have Java 8 and a Docker engine (1.10 or higher) installed. Next install Nextflow with:

curl -s https://get.nextflow.io | bash

Run the example from the repository tree:

./nextflow run Nextflow/workflow.nf -with-docker evolutionar-ygenomics/scalability

To save the graph of the executed workflow, it is sufficient to add the option “-with-dag workflow.pdf.” The same example can also be run without Docker if the required packages have been installed locally following the Bioconda or Guix examples. In this case you can omit the “-with-docker” instruction. To run the example on a compute cluster or in the cloud, it is sufficient to specify a different executor (e.g., sge or awsbatch) in the Nextflow configuration file and ensure that those environments are configured to properly work with the Docker container.

## 4 Discussion

In this chapter we show how to describe and execute the same analysis using a number of workflow systems and how these follow different approaches to tackle execution and reproducibility issues. It is important to assess underlying design choices of these solutions and also to look at the examples we provide online. Even though it may look attractive to opt for the simplest choices, it may be that the associated maintenance burden may be cause for regret later.

The workflow tools introduced in this chapter offer direct integration of software packages. The overall advantage of the bundling software approach is that when software deployment and execution environment are controlled, the logic of the analysis pipeline can be developed separately using descriptive workflows. This separation allows communities to build best practice shareable pipelines without worrying too much about individual system architectures and the underlying environments. An example is the effort by the Global Alliance for Genomics and Health (GA4GH, https://www.ga4gh.org) to develop and share best practice analysis workflows with accompanying container images [45].

In this chapter we also discussed the scaling up of computations through parallelization. In bioinformatics, the common parallelization strategy is to take an existing nonparallel application and divide data into discrete units of work, or jobs, across multiple CPUs and clustered computers. Ideally, running jobs in parallel on a single multicore machine shows linear performance increase for every CPU added, but in reality it is less than linear [46]. Resource contention on the machine, e.g., disk or network I/O, may have processes wait for each other. Also, the last, and perhaps longest, running job causes total timing to show less than linear performance, as the already finished CPUs are idle. In addition to the resource contention on a single machine, the network introduces latencies when data is moved around.

Running the example workflow in the cloud has similar performance and scalability compared to running it on a local infrastructure, after adjusting for differences in hardware and network speeds. Cloud computing is an attractive proposition for scaling up calculation jobs and storing data. Cloud prices for virtual servers and data storage have decreased dramatically, and the possibility of using spot or preemptible instances (i.e., virtual servers that can be priced down to 70% or 80% the normal price but that can be shut down in any moment by the cloud provider) is making cloud computing solutions competitive for high-performance and scientific computing. Cloud essentially outsources hardware and related plumbing and maintenance. Sophisticated tooling allows any researcher to run software in the cloud. We predict an increasing number of groups and institutes will move from large-scale HPC clusters toward tight HPC cluster solutions that can handle continuous throughput with burst compute in the cloud.

Reproducibility is a prime concern in science. Today several solutions are available to address reproducibility concerns. Systems such as Docker and Singularity are built around bundling binary applications and executing them in a container context. Advanced package managers such as Conda or Guix allow the user to create separate software environments where different application versions can be deployed without collisions while ensuring control and traceability over changes and dependencies. All these solutions represent a different approach to address the reproducibility challenge while also offering a different user experience and requiring different setups to work properly. For instance, container-based systems such as Docker and Singularity are not always a viable option in HPC environments since they may require updates to the existing computing infrastructure. Also, HPC operating system installations may include kernel versions that do not allow for the so-called user namespaces, a fundamental component among the many kernel features that together allow an application to run in an isolated container environment. Another downside of containers is that it is hard to assess what is in them—they act like a black box. When creating containers with above Docker scripts, it depends on the time they are assembled what goes in. A Debian or Conda update between creating containers, for example, may include a different software version therefore a different dependency graph. Only GNU Guix containers provide a clear view on what is contained.

Containers provide isolation from the underlying operating system. On HPC environments it may be required to run software outside a container. While applications built with Guix or Conda can be run in isolation when container support is available, they do not require these features at runtime. As a package manager Conda, neither depends on container features nor on root privileges, but it pays for this convenience with a lack of both process isolation and bit-reproducibility [47]. GNU Guix, meanwhile, provides the most rigorous path to reproducible software deployment. In order to guarantee that packages are built in a bit-reproducible fashion and share binary packages, Guix requires to store packages in the directory /gnu/store. There are several work-arounds for this; one of them is by using containers, and another is by mounting /gnu/ store from a host that has built privileges for that directory. A third option is to build packages targeted at a different directory, but this loses the bit-reproducibility and the convenience of binary installs. A fourth option is to provide relocatable binary installation packages that can be installed in a user available directory, similar to what Bioconda does. Such packages exist for sambamba, gemma, and the D-compiler.

Finally, each combination of these packaging and workflow solutions occupies a slightly different region in the solution space for the scalability and reproducibility challenge. Fortunately, the packaging tools can be used next to each other without interference, thereby providing a wealth of software packages for bioinformatics. Today, there is hardly ever a good reason to build software from source.

## 5 Questions

1. Using one of the packaging or container systems described (e.g., Conda, Guix, or Docker), prepare a working environment to run the examples. Now try to run the workflows using the tools presented and appreciate the different approaches to execute the same example.
2. Compare the different syntaxes used by the tools to define a workflow and explore how each tool describes the processes and the dependencies in a different way.
3. Use the Amazon EC2 calculation sheet, and calculate how much it would cost to store 100 GB in S3, and execute a calculation on 100 “large” nodes, each reading 20 GB of data. Do the same for another cloud provider.

## Data Availability

All included software, scripts, and Docker images are based on free and open-source software and can be found at https://github.com/EvolutionaryGenomics/scalability-reproducibility-chapter.

## Box 1 Understanding Parallelization

Parallel computing is related to concurrent computing. In parallelized computing, a computational task is typically broken down in several, often many, very similar subtasks that can be processed independently and whose results are combined afterward, upon completion, i.e., a simple scatter and gather approach. In contrast, in distributed computing, the various processes often do not address related tasks; or when they do, the separate tasks may have a varied nature and often require some interprocess communication during execution. The latter is also a hallmark of supercomputing where compute nodes have high-speed connections.

In the bioinformatics space, we usually discuss embarrassingly parallel computing which means similar tasks are distributed across multiple CPUs without interprocess communication. This can be among multiple cores within a single processor, a multiprocessor system, or a network of computers, a so-called compute cluster.

Even so, parallel multicore programming easily becomes complex. Typically, parallel programming has to deal with extra data and control flow; it has to deal with deadlocks, where depending tasks wait for each other forever and, with race conditions, where tasks try to modify a shared resource (e.g., a file) at the same time resulting in a loss of data or an undetermined condition. This introduces additional complexity in software development, bug hunting, and code maintenance. Typically it takes more time to debug such code than to write it.
